# Aggressive interactions influence cognitive performance in Western Australian magpies

**DOI:** 10.1098/rspb.2024.0435

**Published:** 2024-06-05

**Authors:** Elizabeth M. Speechley, Benjamin J. Ashton, Alex Thornton, Stephanie L. King, Leigh W. Simmons, Sarah L. Woodiss-Field, Amanda R. Ridley

**Affiliations:** ^1^ Centre for Evolutionary Biology, School of Biological Sciences, University of Western Australia, Perth, Western Australia 6009, Australia; ^2^ School of Natural Sciences, Macquarie University, Sydney, New South Wales 2109, Australia; ^3^ Centre for Ecology and Conservation, University of Exeter, Penryn TR10 9FE, UK; ^4^ School of Biological Sciences, University of Bristol, Bristol BS8 1TQ, UK

**Keywords:** social intelligence, aggression, social complexity, cognition, social network, repeatability

## Abstract

Extensive research has investigated the relationship between the social environment and cognition, suggesting that social complexity may drive cognitive evolution and development. However, evidence for this relationship remains equivocal. Group size is often used as a measure of social complexity, but this may not capture intraspecific variation in social interactions. Social network analysis can provide insight into the cognitively demanding challenges associated with group living at the individual level. Here, we use social networks to investigate whether the cognitive performance of wild Western Australian magpies (*Gymnorhina tibicen dorsalis*) is related to group size and individual social connectedness. We quantified social connectedness using four interaction types: proximity, affiliative, agonistic and vocal. Consistent with previous research on this species, individuals in larger groups performed better on an associative learning task. However, social network position was also related to cognitive performance. Individuals receiving aggressive interactions performed better, while those involved in aggressive interactions with more group members performed worse. Overall, this suggests that cognitive performance is related to specific types of social interaction. The findings from this study highlight the value of considering fine-grained metrics of sociality that capture the challenges associated with social life when testing the relationship between the social environment and cognition.

## 1. Introduction

Cognition is defined as the mental processes through which animals collect, retain and use information from their environment to guide their behaviour [1]. Various theories have been proposed to explain the evolution of cognition. The hypothesis that has received arguably the most empirical attention is the social intelligence hypothesis (SIH), which predicts that cognition in social species has evolved owing to the cognitive requirements of living in complex social groups [2–5]. It is hypothesized that social animals face cognitively demanding challenges, including maintaining and coordinating multiple relationships, monitoring group members and recognizing suitable cooperative partners [2–5], which select for greater cognition. Accordingly, the SIH predicts a positive relationship between the social environment and cognition [2–4].

The majority of research has tested the sociality–cognition relationship from an evolutionary perspective, by comparing measures of neuroanatomy or cognitive performance to measures of sociality between species (including primates [6], non-primate mammals [7], birds [8–10], fish [11], reptiles [12] and invertebrates [13]). However, an increasing number of studies are taking an intraspecific approach to the study of cognition [14,15], investigating how the social environment may influence individual cognitive performance. Many of these studies have found that performance in cognitive tests, designed to quantify associative [16] and reversal learning [13], inhibitory control [7], innovation [17], and spatial learning and memory [9], is positively correlated with sociality metrics such as group size [7,9,16,17] and rearing group size [11,13]. However, there are also a number of studies finding no support for the predicted relationship between sociality and cognition [12,18–20].

Despite the premise that dealing with social challenges is cognitively demanding [2–4], relatively few studies have explored explicitly how the type and degree of social interactions influence cognitive performance. Group size is a commonly used sociality metric that corresponds to the largest potential number of conspecifics with which an individual interacts [21]. However, this assumes that individuals within the same group will all experience the same social pressures, when realistically, individuals in social groups may not be interacting equally [22]. Furthermore, more individuals do not necessarily equate to greater informational processing (cognitive) demands, as individuals may not necessarily differentiate between individual conspecifics [23]. Social network metrics are increasingly being used to quantify the social environment experienced by individuals because they capture the diversity of relationships within a group [23]. Social network studies use a variety of sociality metrics, including physical proximity [24], affiliation [25], aggression [26] and vocal interactions [27]. However, few studies have explored social network metrics when relating sociality to cognitive performance, and even fewer explore multiple social network metrics within the same study [28]. The incorporation of multiple social interaction types may give a more comprehensive insight into which social challenges may be impacting individual cognition, and quantifying the repeatability of cognitive performance and social network metrics allows us to determine the robustness of the observed relationship.

Here, we explore the relationship between associative learning (a domain-general cognitive trait that can be used in social contexts [29]) and multiple components of sociality in Western Australian (WA) magpies over consecutive years. Specifically, we explore the relationship between cognitive performance and several network types designed to quantify social connectedness including affiliative, agonistic and vocal interactions and physical proximity. Affiliative and agonistic interactions, as well as physical proximity, are commonly used to quantify social connectedness and are predicted to quantify multiple aspects of social life, including affiliation, conflict and social tolerance [24]. Additionally, since magpies are a highly vocal species [30], we also include vocalizations as a proxy for social interactions. We predict that individuals who are involved in more interactions (strength), with more individuals (degree) will perform better in an associative learning task. Additionally, we predict that where these interactions are more differentiated (coefficient of variation, COV) individuals will perform better in associative learning owing to the demands of remembering, recognizing and responding appropriately to multiple relationships and subsequently optimizing behaviour to the available social information.

## 2. Methods

### (a) Study species and site

WA magpies are a 250–370 g passerine, living in territorial, cooperative groups varying in size between 3 and 12 adults [31,32]. Groups in our study population are located in urban territories of open grassland and parkland in Guildford (31°89′S, 115°96′E) and Crawley (31°98′S, 115°81′E), WA [16]. During data collection, our study population consisted of 18 groups with an average group size of 5.59 ± 0.6 adults (ratio of adults to fledglings 3.13 : 1). The entire study population consists of approximately 80–120 individuals per year. Although WA magpies live in cooperative social groups, we have not observed a consistent dominance hierarchy within groups (personal observation E.M.S. & A.R.R. 2014-2021) [33].

The study population has been monitored regularly since 2014. Individuals are habituated to observers, allowing close behavioural observation and presentation of cognitive tests within 5 m. Individuals are identifiable via coloured and metal bands or by unique scarring or plumage aberrations. Previous research on this population has shown that cognitive performance does not differ between ringed and unringed individuals [34]. Males and females are sexually dimorphic and thus sex is distinguishable by plumage [16]. Sex is known for all adult birds once they reach adult plumage (sex-specific plumage emerges at ~2–3 years old).

### (b) Associative learning

Cognitive performance was measured via associative learning tests. Associative learning is a domain-general trait that is likely to be highly ecologically relevant [35] as it allows individuals to make predictable associations between cues in the environment, such as associating a specific vocalization or posture with an aggressive intent to avoid an incoming attack [29,35]. Associative learning also allows animals to learn the value of associating with different social partners [36]. Furthermore, previous work on this species has shown that performance in associative learning, reversal learning, inhibitory control and spatial memory are correlated [16]; that associative learning performance is highly repeatable [16,37]; and that cognitive performance does not improve according to test number or order when presented with casually identical, visually distinct tests [38]. Therefore, associative learning tests represent a robust measure of an individual’s cognitive performance in this species.

**Figure 1.**
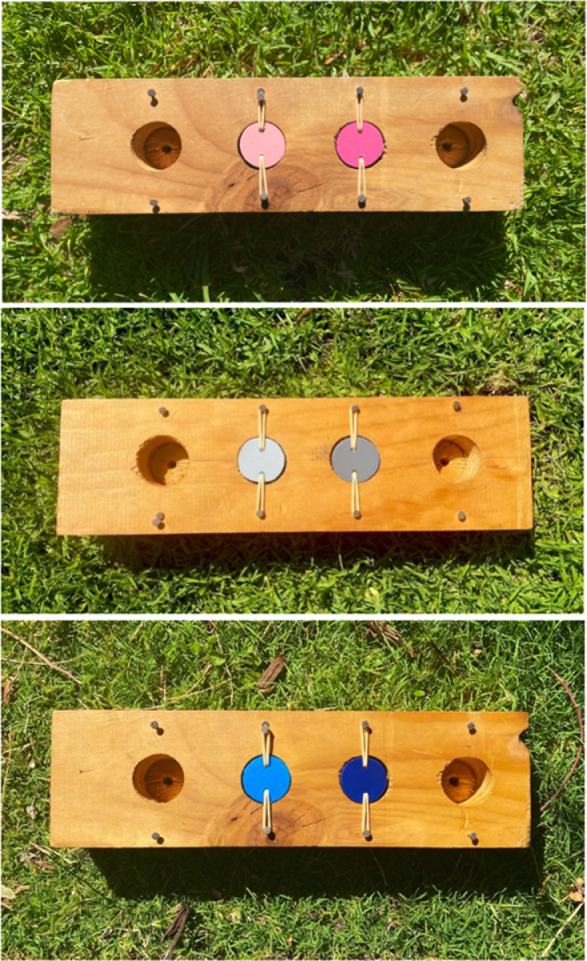
Associative learning array with the colour combinations presented during the three testing periods from 2020 to 2021.

Cognitive testing was conducted using an array of causally identical but visually distinct associative learning tests (figure 1) from March to April 2020, November to December 2020 and March to April 2021 from 5:00 to 11:00. The associative learning array consisted of a wooden grid (31 cm × 9 cm × 4 cm) containing two equidistant wells (3.2 cm diameter × 1.5 cm deep, 3.5 cm apart). Wells were covered with polyvinyl chloride (PVC) lids held in place by elastic bands threaded through drilled holes in the lids and fastened to the sides of the well, allowing the lids to swivel when pecked. The Polyvinyl chloride (PVC) lids were painted with two different shades of a single colour, rather than two distinct colours, to limit the possibility that pre-existing colour biases influence performance [39,40]. To prevent any potential effects of prior learning on performance, the chosen colours had not previously been used in associative learning tests on the individuals tested in this study [16]. The colour combination was also changed during each testing period; specifically, individuals were presented with a pink colour combination in the non-breeding season (March to April 2020), a grey colour combination in the breeding season (November to December 2020) and individuals were presented with either pink or blue in the second non-breeding season (March to April 2021) based on which colour combination they had not been exposed to previously. For example, if an individual was not tested in the non-breeding season in 2020, we were able to test them with the pink colour combination in the non-breeding season 2021 (figure 1). Additionally, as some individuals in the population have been exposed to other colour combinations in previous studies (such as blue, green and purple [16,37]) we made sure that no individual was presented the same colour again in the current study.

Prior to all presentations, both wells were rubbed with cheese to reduce the possibility that olfactory cues may be used to locate the rewarded well. Prior to each trial, the rewarded well was baited, out of sight from the focal individual, with two to three small pieces of mozzarella cheese (~2 cm each). During each trial, the task was placed approximately 5 m in front of the subject. At the beginning of the first trial, one colour shade was chosen, using pseudo-randomization, as the rewarded colour for each individual for the duration of the experiment. The test subject was allowed to search both wells during the first attempt to demonstrate that only one well contained a reward, but in all subsequent trials, they were only allowed to search one well before the apparatus was removed to ensure that there was a cost associated with an incorrect choice. To ensure that the reward was associated with the colour shade, and not the side of the board, the position of the rewarded well was also pseudo-randomized so that it was not on the same side of the array for more than three consecutive trials. The test subject had a minimum of a 1 min interval between trials. As the inter-trial interval was very consistent (~1 min), the focal bird was usually still present after rebaiting. Rebaiting occurs by the experimenter turns their back to the focal individual and rebaiting the array out of sight. Following Ashton *et al*. [16], an individual was considered to have passed the test when they selected the rewarded colour in 10 out of 12 consecutive trials (representing a significant deviation from random binomial probability). If the subject did not complete the task in 1 day, tests were continued the following day until the individual passed the test. All individuals were tested in social isolation (with conspecifics at least 10 m away) [16]. As magpies frequently forage far apart (10–20 m), there was ample time for an individual to complete the task before another individual approached [16]. Additionally, a second observer walked with the rest of the group to ensure conspecifics were not approaching or watching the focal bird.

### (c) Additional explanatory factors

The order tested in the group (the number of birds in the group that were tested before the focal bird) was recorded during testing, to account for any potential social learning, as well as any anti-predator behaviours. We measured the average ambient temperature at the time of testing (°C) as previous research has found that cognitive performance in this species declines owing to heat stress above 32°C [41]. Furthermore, all testing was conducted at temperatures below 30°C when individuals were not showing signs of heat dissipation, such as panting or wing spreading (mean temperature during testing in March to April 2020 18.74 ± 0.52°C, range: 12–25.5°C; November to December 2020 16.09 ± 0.31°C, range: 10.8–22°C; March to April 2021 20.49 ± 0.45°C, range: 14–27.4°C).

We also measured several proxies of motivation and body condition. First, we regularly collect data on body mass. This was performed by encouraging individuals to hop onto a top-pan scale (Ohaus Challenger series, 1000 ± 1 g) using a small food reward (<1 g mozzarella cheese) [16]. Second, we conducted weekly foraging focal observations to calculate foraging efficiency. Foraging focal observations lasted 10 min per individual and were recorded using a customized program in Cybertracker [42]. When a successful foraging attempt was made, the approximate size of the prey was recorded according to size categories relative to magpie bill length and converted to grams as outlined in Edwards *et al*. [31]. Individual foraging efficiency was calculated as the amount of food (in grams) caught per minute of time spent foraging [31]. Body mass and foraging focals were collected during the morning (5:00–11:00). Body mass was measured the morning prior to cognitive testing and foraging focals were conducted within a week of cognitive testing, and where possible multiple foraging focals were collected and averaged (mean: 59.36 focal minutes per individual). Finally, we also recorded neophobia (latency to contact the task) as a proxy of motivation. Neophobia was defined as the time elapsed between approaching within 1 m of the apparatus and first making contact with the apparatus.

### (d) Social networks

Social network observations were conducted on a weekly basis from February to June 2020 and 2021 (non-breeding seasons) and August to October 2020 and 2021 (breeding seasons) with 10 observations per group per season (40 total per group, except for one group, where *n* = 30). Social network observations were only conducted on groups where all individuals were identifiable. Each observation was conducted over 90 min at varying times from 5:00 to 11:00. Interactions were recorded ad libitum on a Samsung Galaxy Tablet A6 using Cybertracker [42].

All-occurrence sampling was used to collect social interaction data on affiliative, agonistic and vocal behaviour. Affiliative interactions included allopreening and play (although instances of allopreening were rare and thus the affiliation interactions primarily represent play). Agonistic interactions consisted of fighting, chasing, pecking, splays (a dominance display) and physical attacks. Vocal interactions were restricted to vocal choruses, because this was the only two-individual vocal with an identifiable initiator and receiver that occurred frequently enough to record. The chorus is a common vocalization in magpies that is given in a number of contexts, including during competitive inter/intra-group interactions, group disturbances (e.g. predator events) and mating and social cohesion (personal observation E.M.S. 2020-2021). Where possible, the actor (initiator) and receiver of all social interactions were identified.

During the collection of social interaction data, proximity was estimated using scan sampling, recording the approximate distance (metres) between each individual in the group every 15 min. Group sampling was chosen as opposed to focal sampling because it maximizes the number of potential interactions recorded during a single sampling event and has been shown to perform better than focal sampling methods [43]. To ensure that proximity was recorded accurately and consistently, two observers (E.M.S. and S.L.W.-F.) estimated distances ranging from 1 to 120 m (representing the range of distances recorded during the observations) with 89% and 88% accuracy, respectively.

### (e) Statistical analysis

#### (i) Social networks

All network analyses were conducted in R v. 4.2.1 [44]. Each social group was analysed separately for each time period (i.e. non-breeding season 2020 *n*_(network observation)_ = 12, non-breeding season 2021 *n*_(network observation)_ = 13, breeding season 2020 *n*_(network observation)_ = 13, and breeding season 2021 *n*_(network observation)_ = 13), with a separate network for each interaction type (affiliative, agonistic, vocal and proximity). Proximity data were converted into association matrices using the Simple Ratio Index (SRI) [45] via the *asnipe* package [46]. The SRI is an estimate of the proportion of time two animals spend together (0 for pairs of animals never observed together; 1 for pairs always seen together). To determine an appropriate cut-off for proximity networks, we ran proximity networks at four different cut-offs, restricting datasets to proximity measures within 1, 2, 5 and 15 m. Since networks based on 1 m proximity showed the most variation in relationship strength (indicated by the highest COV values and permutations significantly different to random), a proximity cut-off of 1 m was used for all analyses (electronic supplementary material, table S1). Interaction data were converted into directed network matrices (agonistic and vocal) or undirected network matrices (affiliative) using custom-written R scripts [47]. Permutations were run for each network by shuffling the data in the interaction matrices (controlling for the number of interactions per actor in directed networks) and recalculating the COV 1000 times. The observed COV was then compared with the random distribution to confirm that the relationship differentiation in the observed networks was significantly different to that expected by chance [46].

From these matrices, two node-level network measures were calculated (degree and strength) using the *sna* package [48]. Each of these metrics quantified a different aspect of social centrality. The degree is the number of conspecifics the individual interacts with, whereas strength (also referred to as weighted degree centrality) is the sum of the weighted edges connected to an individual and represents how well connected an individual is in its network [49]. For each network, we also calculated the COV, which represents the number of differentiated relationships or interactions within a network (smaller COVs indicate that individuals associate or interact more evenly with group members, while higher COVs indicate more heterogeneous associations or interactions). All networks were treated as weighted networks, which are especially useful in small groups where the frequency of connections is more informative than their presence [50].

For directed networks (agonistic and vocal) which involve an actor and a receiver, in-degree (the number of conspecifics the individual receives aggressive interactions from), out-degree (the number of conspecifics the individual initiates aggressive interactions toward), in-strength (the frequency of interactions the individual received) and out-strength (the frequency of interactions the individual initiates) were also calculated [49]. As groups vary in size, node-level network measures were normalized to allow for comparisons between groups. That is, node strength for each individual was divided by the highest node strength within that group, thus, scaling node strength between 0 and 1 (where individuals with a score of 1 showed the most social connectedness in that group).

Finally, a sensitivity analysis was conducted to determine the minimum number of observations required for each individual in order for them to be included in the analysis. Based on this output, only individuals with a minimum of 10 focal observations were included in the final analysis (electronic supplementary material, figure S1), allowing a good balance between improved accuracy of network metrics and not excluding too many focals.

#### (ii) Predictors of cognitive performance

All statistical analyses were conducted in R v. 4.2.1 [44]. The data were initially checked to confirm they met model assumptions, including the normality of residuals, presence of outliers and dispersion using the *DHARMa* package [51].

Affiliative observations were omitted from the analysis owing to low sample size: affiliative interactions were rarely observed between adults (see Results). Proximity metrics were also omitted from the analysis because 46 out of 51 networks were not significantly different from random (electronic supplementary material, table S2).

Prior to the main analysis, we ran a series of exploratory generalized linear mixed models (GLMMs) with a Poisson distribution using the *lme4* package [52]. These exploratory analyses were designed to test potential factors that might confound cognitive performance, including motivational factors (neophobia, body mass and foraging efficiency) and physical/environmental factors (the assigned colour, shade of rewarded well, average ambient temperature and order tested) as explanatory terms. Associative learning score (number of trials taken to pass the task) was the response term and group identity was included as a random term. Any terms that were deemed to influence cognitive performance were then included in the main analysis to account for their effect on cognitive performance [53].

To test the hypothesis that the social environment is related to associative learning performance, we ran a series of GLMMs with a Poisson distribution as described above. We paired cognitive testing conducted during March to April 2020 and 2021 to the social measures taken during February to June 2020 and 2021. Cognitive testing during November to December 2020 was linked to the social measures taken during August to October 2020 (as no cognition testing was conducted from November to December 2021, the social network measures from August to October 2021 were used solely for repeatability analysis, described below). Cognitive performance was measured as the number of trials taken to pass the task. Explanatory terms included current group size as well as social network metrics including normalized degree, in-degree, out-degree, strength, in-strength, out-strength, and coefficients of variation for the agonistic and vocal networks (see electronic supplementary material for glossary of terms). All explanatory variables were tested for a correlation with one another prior to being included in the model (variance inflation factor VIF >2 for pairs of continuous predictors was considered a high level of correlation) [54]. As VIF scores were greater than 2 for agonistic strength and out-strength, agonistic strength was removed as an explanatory term. Similarly, as VIF >2 for vocal strength and out-strength, vocal strength was removed as an explanatory term. In each case, the weaker predictor of the two terms was removed. Individual identity, group identity and year were included as random terms in all models owing to repeated testing.

Prior to testing in 2020 several of our groups had major group splits. Therefore, we also ran a subset GLMM analysis using individuals for which group size was recorded for at least 4 years prior to 2020, or individuals for which we knew their group size over the entirety of their life. We then filtered this dataset to only include individuals for which group size had remained stable over this 4-year period (removing groups that had changed in size by more than three individuals; electronic supplementary material, table S3). This GLMM was run as described above with associative learning score as the response term and group size as the explanatory term. Individual identity and group identity were included as random terms owing to repeated testing.

We determined the best model using the model selection process outlined by Harrison *et al*. [55], where terms were ranked in order of their Akaike information criterion score corrected for small sample sizes (AICc). If a model was within two ΔAICc units of the best model (lowest AICc) and the effects had 95% CIs that did not intersect zero [56], it was included in the top model set. Sample sizes reflect the datasets after removing missing values.

#### (iii) Repeatability

Repeatability analyses were conducted using the *rptR* [57] package. The data were initially checked to confirm they met model assumptions (as above) using the *DHARMa* package [51]. A GLMM using a quasi-Poisson distribution (corrected for overdispersion) was used to determine the repeatability of individual cognitive performance for the full dataset. We also ran a repeatability analysis on data collected in 2020 only (Gaussian distribution). As cognitive scores were only collected in the non-breeding season 2021, analysing 2020 alone allows a direct comparison of cognitive performance in the breeding versus non-breeding season. GLMM estimates with a Gaussian distribution were used to determine the repeatability of social network metrics (agonistic and vocal degree, in-degree, out-degree, strength, in-strength, out-strength and COV) in the full dataset and the 2020-only subset. In each model, individual identity was included as a random factor, except for COV, which is a group-based metric and thus group identity was included as a random factor.

## 3. Results

### (a) Associative learning

We tested 53 individuals from 16 groups between March and April 2020, 62 individuals from 16 groups between November and December 2020, and 49 individuals from 16 groups between March and April 2021, with a mean number of trials to reach the criterion of 19.47 (range: 10–42), 16.48 (range: 10–39) and 13.96 (range: 10–34) per individual, respectively. A total of 37 individuals were tested twice and 18 were tested three times on visually distinct, but casually identical tasks (figure 1). The subset of individuals with a complete set of explanatory factors and social network metrics used for GLMM analysis comprised 82 cognitive tests on 47 individuals from 9 groups. Group sizes for tested individuals ranged from 3 to 10 adults per group (mean, 6.44 ± 0.54). Other studies have used social network analysis with similar-sized groups [8,58].

### (b) Social network observations

A total of 765 h of social network observations were conducted (180 h on 12 groups from February to June 2020, 195 h on 13 groups during each of the following time periods: August to October 2020, February to June 2021 and August to October 2021). Affiliative interactions mostly consisted of play, with only one instance of allopreening. Play was rarely observed between adults (0.02%, 8 out of 412 instances of play). Agonistic interactions were observed for territory defence, mate/nest guarding and as a begging reprimand for juveniles and thus were more frequent during the breeding season (electronic supplementary material, figure 2*a*). Aggression often occurred between adults and juveniles and was primarily within-sex interactions (electronic supplementary material, figure S3*a*). Vocal interactions were the most frequently observed interactions, with up to 1277 choruses recorded per group during the non-breeding season 2020 (average of 613 choruses recorded per group, range: 152–1277; electronic supplementary material, figure S3*b*). Unlike agonistic interactions, vocal interactions were common in both the breeding and non-breeding seasons (electronic supplementary material, figure S2*b*).

### (c) Predictors of cognitive performance

In our exploratory analysis, body mass and order tested performed better than the null model and thus were included in the main analysis (electronic supplementary material, table S4). We found no relationship between foraging efficiency and group size, indicating that competition for food is not greater in larger groups (estimate: −0.23, s.e., 024, CI: −0.70/0.24, *p* = 0.34).

**Figure 2.**
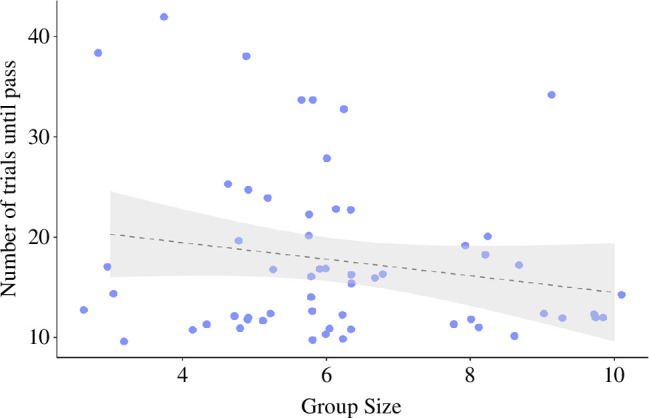
Relationship between group size and the number of trials taken to complete the associative learning test (*n* = 58 cognitive tests on 33 individuals from 10 groups). A lower number of trials indicates better performance in the test. Points are raw data (jittered for visibility). The shaded area represents the 95% CIs.

There was a significant relationship between associative learning score and group size when analysing individuals who had not experienced major group size changes over a 4-year observation period (figure 2). Specifically, we find that individuals in larger groups solve the associative learning task in fewer trials than individuals in smaller groups (estimate: −0.07, s.e.: 0.03, CI: −0.13/−0.03, *p* = 0.04; figure 2).

**Figure 3.**
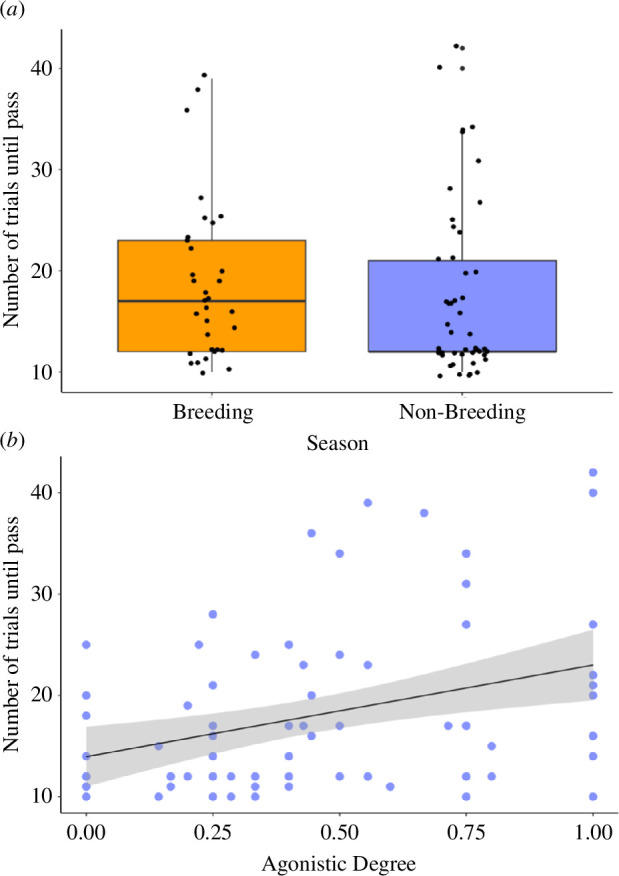
Relationship between (*a*) season and (*b*) agonistic degree and the number of trials taken to complete the associative learning test (*n* = 82 cognitive tests on 47 individuals from 9 groups). A lower number of trials indicates better performance in the test. (*a*) Points are raw data jittered for visibility. The box represents 50% of the variation and the whiskers represent the 5th and 95th percentiles. The horizontal line represents the median. (*b*) Points are raw data (jittered for visibility). The shaded area represents the 95% CIs.

**Figure 4.**
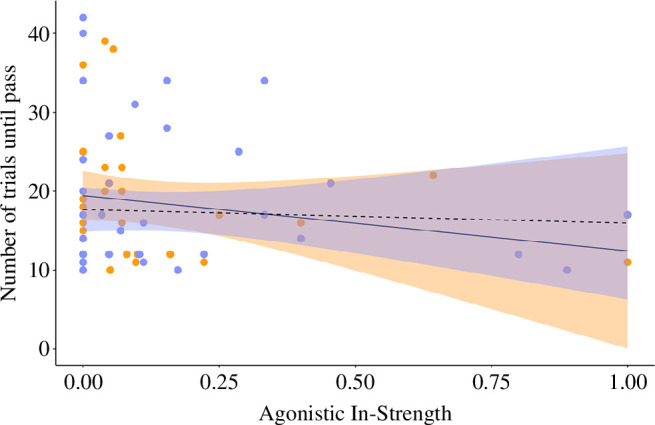
Relationship between agonistic in-strength and the number of trials taken to complete the associative learning test showing an interaction with season (*n* = 82 cognitive tests on 47 individuals from 9 groups). A lower number of trials indicates better performance in the test. Points are raw data (jittered for visibility), solid fitted line and orange points represent the breeding season and broken line and purple points represent the non-breeding season. The shaded area represents the 95% CIs (orange for breeding season and purple for non-breeding season).

Season, agonistic degree and agonistic in-strength were the strongest predictors of individual cognitive performance (table 1; see electronic supplementary material, table S5 for full model selection output). Individuals involved in aggressive interactions with more group members performed worse in associative learning tests during both seasons (figure 3). Additionally, individuals who frequently received aggressive interactions during the breeding season performed better at associative learning tests compared with those who received less aggression (figure 4). Cognitive performance was not related to any other network measures, nor was it related to any of our proxies of motivation (body mass, foraging efficiency or neophobia/latency to contact the task). We found no correlation between any of our agonistic network measures and our proxies of motivation (VIF <2).

**Table 1.** Top model set of candidate terms affecting performance in the associative learning task using model selection.

		AICc	△AICc
null model		585.79	23.31
top models			
season + agonistic degree		562.48	0
season × agonistic in-strength		564.08	1.60

effect sizes of top model	effect	s.e.	**95% CI**
intercept	2.64	0.18	2.28/3.00
season (non-breeding)	0.23	0.07	0.09/0.37
agonistic degree	0.16	0.04	0.09/0.23
intercept	2.63	0.19	2.25/3.00
season (non-breeding)	0.22	0.08	0.07/0.14
agonistic in-strength	−0.23	0.06	−0.35/−0.12
season (non-breeding) × agonistic in-strength	0.29	0.08	0.14/0.45

*Notes:* AICc values are provided for models within 2 ΔAICc of the top model and with predictors whose 95% CIs do not intersect zero. Coefficient estimates (effect), standard error (s.e.) and 95% CIs are given below the top model set. *n* = 82 cognitive tests on 47 individuals from 9 groups. individual identity, group identity and year were included as random terms; see electronic supplementary material, table S5 for full model selection output.

### (d) Repeatability

Individual cognitive performance was significantly repeatable in the full dataset (electronic supplementary material, table S6). We also analysed 2020 scores only. As cognitive scores were only collected in the non-breeding season of 2021, analysing only 2020 scores allows a direct comparison of cognitive performance in the breeding versus non-breeding season [30]. Analysis showed that cognitive performance was not repeatable between the breeding and non-breeding season (electronic supplementary material, table S6). When using the full dataset, all social network metrics were highly repeatable across seasons and years, apart from vocal in-degree (electronic supplementary material, table S7*a*). The 2020 subset also showed that all network metrics were repeatable (across season), apart from agonistic out-strength and vocal in-degree, which complements the finding that aggression is more frequent in the breeding season (electronic supplementary material, table S7*b*).

## 4. Discussion

We quantified group size and individual social network position using a range of social network metrics, with the aim of exploring whether the challenges associated with the social environment influence cognitive performance. We find that individuals in larger groups solve the associative learning task in fewer trials, and that task performance is related to agonistic degree, whereby individuals involved in aggressive interactions with more group members performed worse in cognitive tests. Additionally, we find that the individuals who received aggressive interactions more frequently during the breeding season perform better in cognitive tests compared with those who received less aggression.

Here, we provide evidence that cognitive performance is linked with sociality, with individuals in larger groups performing better in associative learning tests than individuals in smaller groups. This supports previous work on this population showing that adults and fledglings in larger groups perform better in cognitive tests [16,59]. Combined, these studies suggest that the social environment plays an important role in shaping cognitive performance, supporting recent research in other species [7,28,60]. We also find that certain social interactions are associated with poorer cognitive performance; specifically, individuals involved in aggressive interactions with more group members (degree) perform worse in cognitive tests. One possible explanation is that individuals involved in aggression, regardless of whether they receive or initiate aggression, may have less time or energy to attend to the cognitive task (although it should be noted that no relationship was detected between latency to interact with the task and aggression, and all tests were conducted in social isolation). However, when we interpret this result in conjunction with the frequency of aggression (in-strength and out-strength) we find that individuals receiving aggression more frequently (in-strength) perform better at the associative learning test, particularly during the breeding season. Although not in the top model set, we also find that individuals initiating aggression more frequently (out-strength) perform worse in the cognitive tests (note that season × agonistic out-strength was the next top model behind the two top models reported in results and substantially better than the null, electronic supplementary material, table S5). These combined findings suggest that individuals receiving high amounts of aggression may have a greater requirement to make associations between cues in their environment (e.g. vocal and postural cues) and a potential aggressive threat. Additionally, individuals who frequently initiate aggression with conspecifics may have lower social competence [61] and lower self-control than those who are less aggressive [62]. The previous work on carrion crows (*Corvus corone corone*) similarly argued that aggressive individuals would perform worse in cognitive tests [26]. Collectively, our results suggest that aggressive interactions play an important role in cognitive performance in magpies.

Our finding of a relationship between aggression and cognition may support the ‘necessity drives innovation’ hypothesis, which suggests that individuals will invest more in finding solutions to new problems if they are unable to monopolize resources [63]. Thus, it is possible that more aggressive individuals can acquire more resources, whereas less aggressive individuals need to invest more time in finding novel solutions if they are less able to monopolize resources. For instance, in great tits (*Parus major*) competitive ability was negatively correlated with problem-solving performance, whereby poor competitors were better problem solvers [64] (but also refer to [65]). The ‘necessity drives innovation’ hypothesis is further supported by our agonistic in-strength result, where individuals receiving more aggression perform better in an associative learning task. However, there is no evidence that aggression is related to food resource acquisition in magpies, with a food provisioning study finding no relationship between aggression and monopoly of supplemented food [33]. In addition, despite over 10 years of observation on the study population, we have not observed kleptoparasitism events between group members, suggesting aggression does not facilitate the acquisition of food from others. However, as aggression may be related to the monopoly of rich food patches in the environment, we cannot completely rule out the ‘necessity drives innovation’ hypothesis as a potential explanation for our results.

The relationship between cognitive performance and agonistic in-strength may also reflect a response to heightened stress or a personality–cognition trade-off. Previous studies have shown that aggressive individuals tend to have higher levels of corticosterone [66]. Cortisol or corticosterone is secreted by the hypothalamic–pituitary–adrenal axis as a response to stressors [67], and stress and glucocorticoids are known to influence cognitive performance [68]. However, the direction of this relationship is often unpredictable [69]. Furthermore, proactive individuals (bold, aggressive, explorative or active) are often associated with cognitive speed–accuracy trade-offs [70]. Proactive individuals are often quicker to approach a novel object, faster to explore, more aggressive to conspecifics and less flexible in their behaviour [67]. Thus, it is possible that aggression itself represents a motivational confound in this study. However, we find no correlation between neophobia (latency to contact the task) and aggression, and none of our other measures of motivation (body mass, foraging efficiency or latency to contact the test) predict cognitive performance. Ultimately, further investigation is required between other proactive traits and cognition to establish whether a personality-cognition trade-off exists in WA magpies.

Our study also accounts for changes in cognitive performance and social connectedness over multiple seasons and years. Here, we find a positive relationship between cognitive performance and social group size when group size remains stable over a period of 4 years. Additionally, previous research on this population has shown that rearing group size effects cognitive development, whereby fledglings reared in larger group show higher cognitive performance at 200 and 300 days post-fledgling [16,59]. The increased aggressive interactions observed during the breeding season and associated higher cognitive performance in those receiving more aggression may be indicative of cognitive plasticity, whereby cognitive performance changes under different environmental conditions [71]. Although overall rates of aggression increase during breeding, when analysed over multiple years all social network metrics were highly repeatable, establishing a long-term pattern of behaviour. The repeatability of individual social network metrics over multiple years complements existing literature [72,73]. Our results also complement previous work on our study population, which has shown that cognition is repeatable when analysed over multiple years [37,38]. An informative path of future enquiry would be to explore the ontogeny of the social network–cognition relationship to determine whether aggressive interactions are important during cognitive development, and if these are repeatable across a longer time period.

We present one of the few studies that have incorporated multiple measures of social interactions when exploring the relationship between the social environment and cognitive performance. The utilization of social network analysis allowed us to explore the informational challenges associated with interacting with large numbers of individuals or interacting more frequently, beyond simple dyadic interactions. We tested four types of social interaction and found that both affiliative interactions and physical proximity are not robust measures of social interaction in adult magpies. However, affiliative interactions occur regularly in fledglings and such interactions may play a role in the development of cognitive phenotypes. Additionally, most of our proximity networks were not significantly different to random, and thus do not capture meaningful social interactions. Although proximity did not prove a robust proxy for social complexity in this species, it has been proven to be an effective measure in other species of both birds [10] and primates [58]. Ultimately, we find that only agonistic interactions predict an individual’s cognitive performance. This suggests that the relationship between cognition and sociality may depend on the demands associated with specific social interactions.

Overall, we find that cognitive performance is related to agonistic in-strength, whereby individuals receiving more aggression perform better in an associative learning task. We also find that individual cognitive performance is related to agonistic degree, whereby individuals with a greater number of aggressive connections with other group members perform worse in an associate learning task. We have presented a detailed exploration of the relationship between social interactions and cognition over time to determine how cognitive performance may be impacted by various measures of sociality, including social network connectivity, based on multiple interaction types. Our findings highlight the importance of including fine-grained metrics of sociality, which capture the informational challenges associated with the social environment, when investigating the relationship between the social environment and cognition.

## Data Availability

Data are available from the Dryad Digital Repository [47]. Electronic supplementary material is available online at Figshare [74].
